# Visualizing the Osteocyte Lacuno-Canalicular System via a Rapid 10-Minute Silver Nitrate Staining Method

**DOI:** 10.21769/BioProtoc.5784

**Published:** 2026-08-05

**Authors:** Jinlian Wu, Weiwei Dai, Libo Wang

**Affiliations:** 1 Department of Rheumatology and Immunology, Longhua Hospital, Shanghai University of Traditional Chinese Medicine, Shanghai, China; 2 Central Laboratory of Science and Technology, Longhua Hospital, Shanghai University of Traditional Chinese Medicine, Shanghai, China

**Keywords:** Wu–Wang silver nitrate staining method, Ploton silver nitrate staining method, Osteocyte, Lacuno-canalicular system (LCS), Bone

## Abstract

The conventional Ploton silver method employs a high-concentration 50% (w/v; 2.943 mol/L) silver nitrate solution for histological staining and characterization of the osteocyte lacuno-canalicular system (LCS). However, it is limited by prolonged staining times (55 min) and by risks of LCS ultrastructural damage and/or incomplete impregnation. To address these limitations, we developed the Wu–Wang silver nitrate staining method, which uses a 1 mol/L silver nitrate solution under elevated temperature (50–70 °C) to achieve rapid, effective, and high-contrast visualization of the osteocyte LCS within 10 min. We further demonstrate that this novel method enables robust LCS visualization across multiple vertebrate species. Compared with the Ploton method, the Wu–Wang method substantially reduces staining time and overcomes staining limitations inherent to prolonged exposure to concentrated silver nitrate solutions. This rapid and efficient staining method supports more accurate quantitative analysis of LCS morphology and facilitates systematic investigation of osteocyte and LCS morphogenesis, as well as the pathological mechanisms underlying bone and joint disease.

Key features

• A novel rapid method uses 1 mol/L silver nitrate at 50–70 °C, enabling high-contrast LCS staining within 10 min.

• A rapid silver nitrate staining method provides superior, consistent LCS visualization across multiple vertebrate species.

• The novel method serves as a fast, efficient histological tool to investigate osteocytes, LCS, and bone–joint pathology.

## Graphical overview


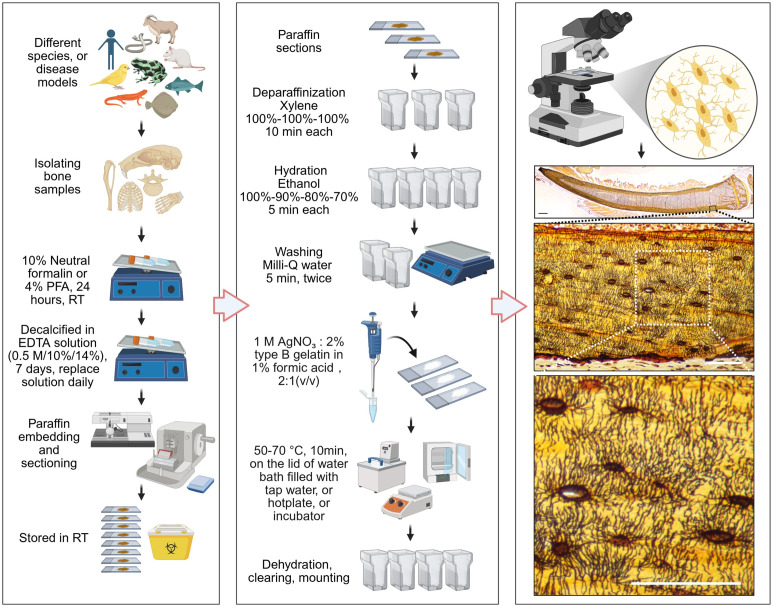


**Workflow of the Wu–Wang silver nitrate staining method for osteocyte lacuno-canalicular system (LCS) visualization.** Created with BioRender (https://BioRender.com).

## Background

Osteocytes, derived from osteoblasts and embedded within the mineralized bone matrix, play an essential role in both bone development and the progression of bone and joint diseases [1–2]. Osteocytes reside in lacunae and extend long cellular processes into an interconnected network of canaliculi, which together constitute the lacuno–canalicular system (LCS) [3–4]. Through LCS architecture, osteocytes function as mechanosensors and signal transducers, thereby regulating bone remodeling and maintaining mineral homeostasis [5–6]. Abnormalities in LCS structure during aging or disease can alter remodeling dynamics and disrupt mineral homeostasis, ultimately contributing to a range of musculoskeletal and degenerative disorders [7–10].

Despite the importance of the LCS, detailed morphological analyses and a comprehensive understanding of LCS development remain challenging, largely due to the lack of rapid, robust, and reproducible histological staining approaches. The currently widely used Ploton silver staining method often yields variable results, with inconsistencies arising from differences in reagent concentration and composition [11–14]. In previous studies [15–16], we showed that using lower silver nitrate concentrations (0.5–1 mol/L) enables effective and clear visualization of the LCS while reducing tissue damage and improving staining adequacy compared with the Ploton formulation (50% w/v; 2.943 mol/L). Nevertheless, the staining time of our optimized method (60 min) remained comparable to that of the Ploton method (55 min), which may limit its practicality and broader adoption. Building on our earlier observations that temperature can facilitate silver staining [16], and informed by literature describing how temperature affects silver-based staining reactions [17–19], we systematically evaluated the effect of incubation temperature on LCS silver nitrate staining [20]. Surprisingly, we discovered and established a rapid and efficient staining condition: incubation with 1 mol/L silver nitrate at 50–70 °C for 10 min [20].

Herein, we present a detailed protocol for this novel histological staining method, including step-by-step procedures and practical notes (see Graphical overview). We further show that this novel method enables rapid, high-contrast, and clear visualization of osteocyte LCS in mouse skeletal tissues as well as in bone specimens from diverse vertebrate species. Widespread adoption and application of this rapid and efficient histological method will advance our understanding of osteocyte and LCS morphogenesis among osteocyte biologists and evolutionary developmental biologists, while simultaneously providing a practical histological tool for investigating the pathological mechanisms of bone and joint diseases in clinical research.

## Materials and reagents

**Biological materials**

1. 8-month-old male C57BL/6J mice (Jiangsu GemPharmatech Co., Ltd.)

2. Domestic pigs (*Sus scrofa domesticus*), domestic black-spotted frogs (*Pelophylax nigromaculatus*), Chinese three-keeled pond turtles (*Mauremys reevesii*), domestic pigeons (*Columba livia domestica*), grass carp (*Ctenopharyngodon idella*), and Asian swamp eels (*Monopterus albus*), obtained from local food markets

3. Zebrafish (*Danio rerio*) and rare gudgeons (*Gobiocypris rarus*), obtained from local aquarium suppliers

**Reagents**

1. Silver nitrate solution, 1 mol/L (Bolinda Technology, catalog number: P1929026)

*Note: Alternatively, commercially available silver nitrate powder can be purchased and used to prepare a 1 mol/L silver nitrate solution with 18.2 MΩ/cm Milli-Q water.*

2. Type-B gelatin (Sangon Biotech, catalog number: A600908–0500)

3. Formic acid (FA) 88% (Sinopharm, catalog number: 10010118)

4. EDTA decalcification solution 0.5 mol/L (Servicebio, catalog number: G1105-500ML)

*Note: Alternatively, commercially available 10% or 14% EDTA decalcification solutions can also be used. Rapid decalcification solutions, such as formic acid–based decalcification solutions, should be avoided because they result in substantially poorer LCS staining quality than EDTA decalcification solutions.*

5. Neutral formalin fixative 10% (Wexis, catalog number: 311010014)

*Note: Alternatively, commercially available 4% paraformaldehyde (PFA) (Servicebio, catalog number: G1101-500ML) can be used.*

6. PBS (Servicebio, catalog number: G2156-1L)

7. Paraffin (Leica, catalog number: 39601006)

8. Ethanol (SinoPharm, catalog number: 10009218)

9. Xylene (SinoPharm, catalog number: 10023418)

10. Neutral balsam mounting medium (Solarbio, catalog number: G8590)

**Solutions**

1. 1% (v/v) FA solution (see Recipes)

2. 2% (w/v) gelatin in 1% FA solution (see Recipes)

3. Silver nitrate staining solution (see Recipes)

**Recipes**

**1. 1% (v/v) FA solution**

**Table:** 

Reagent	Final concentration	Quantity or volume
88% FA	1% (v/v)	1.136 mL
18.2 MΩ/cm Milli-Q water	n/a	98.864 mL
Total	n/a	100 mL

The prepared solution can be stored at room temperature and does not require light protection.

**2. 2% (w/v) gelatin in 1% FA solution**

**Table:** 

Reagent	Final concentration	Quantity or volume
Type-B gelatin	2% (v/w)	2 g
1% FA solution	n/a	100 mL
Total	n/a	100 mL

The prepared solution can be stored at room temperature and does not require light protection. See General note 1.

**3. Silver nitrate staining solution**

**Table:** 

Reagent	Final concentration	Quantity or volume
1 mol/L silver nitrate solution	33.3% (v/v)	0.4 mL
2% (w/v) gelatin in 1% FA solution	66.7% (v/v)	0.2 mL
Total	n/a	0.6 mL

The staining solution should be prepared immediately before use and protected from light throughout the preparation process. See General note 2.

**Laboratory supplies**

1. Slides (Citotest, catalog number: 80312–3161)

2. Cover slides (Citotest, catalog number: 80340–0130)

3. Pipettes (Gilson, models: P1000 and P200)

4. 50 mL tubes (Greiner, catalog number: 227651)

5. Embedding cassettes (Citotest, catalog number: 31050102W)

6. Forceps and scissors

## Equipment

1. High-precision water bath (Jinghong, model: DKB-501S)

*Note: Alternatively, a temperature-controlled incubator with precise temperature regulation or a temperature-controlled hotplate can also be used.*

2. Automatic benchtop tissue processor (Leica, model: TP1020)

3. Tissue embedding station (Kedi, model: KD-BM)

4. Rotary microtomes (Leica, model: RM2135)

5. Histology water bath (Leica, model: Histobath HI1210)

6. Ultrapure water purification system (Millipore, model: Milli-Q Elix^®^ Essential)

7. Nikon microscope (Nikon, model: ECLIPSE 200)

## Procedure

**A. Bone sample collection, fixation, embedding, and sectioning**

1. Bone specimen procurement: Euthanize the laboratory animals (e.g., mice) of the specified age via carbon dioxide inhalation. When using food-derived material, obtain edible animal-derived bones from local food markets sourced from animals already slaughtered by vendors.

2. Dissection: After euthanasia, dissect the required bones (e.g., femur, tibia, vertebrae, or other bones of interest) using scissors and forceps. For edible animal meat–derived bones obtained from local food markets, isolate the target bone portion as quickly as possible using an electric saw or bone scissors. Carefully remove surrounding muscle and adipose tissue from the bones as completely as possible using scissors and forceps in 4 °C pre-chilled PBS. After tissue removal, rinse the bones in 4 °C pre-chilled PBS to remove blood and any residual tissue debris.

3. Fixation: Transfer the bones to 10% neutral buffered formalin or 4% PFA. Ensure that the fixative volume is at least 10-fold greater than the tissue volume. See General note 3. Fix at room temperature on a vertical shaker for 24 h.

4. Washing: After fixation, wash the bones on the vertical shaker at room temperature using at least a 10-fold volume of Milli-Q water relative to the bone volume for 6 cycles, 10 min per cycle, to remove residual fixative. See General note 3.

5. Decalcification: Decalcify the bones using at least a 10-fold volume of 0.5 mol/L EDTA (or 10%/14% EDTA, pH 7.4) relative to the bone volume. See General note 3. Perform decalcification at room temperature on a vertical shaker for 7 days, replacing the EDTA solution every 24 h.

6. Post-decalcification washing: Following decalcification, wash again using at least a 10-fold volume of Milli-Q water relative to the tissue volume on the vertical shaker at room temperature for 6 cycles, 10 min per cycle, to remove residual EDTA. See General note 3.

7. Dehydration: Dehydrate the bones through a graded ethanol series (70%, 80%, and 90% ethanol) for 1 h each, followed by three sequential incubations in 100% ethanol for 1 h each to ensure complete dehydration using an automatic benchtop tissue processor.

*Note: During the three steps of dehydration, clearing, and paraffin infiltration—comprising a total of 12 solvent baths—we perform all processing using an automatic benchtop tissue processor. In this instrument, each bath contains at least 500 mL of the corresponding solvent (e.g., ethanol, xylene, or melted paraffin) to ensure adequate dehydration, clearing, and paraffin infiltration.*

**Caution:** Ethanol is flammable and poses a severe fire hazard. Work only in designated areas and a fume hood. Avoid direct contact with reagents. Always wear gloves and other appropriate personal protective equipment (PPE).

8. Clearing: Clear the bones in 50% ethanol–50% xylene (v/v) for 1 h, followed by two sequential incubations in 100% xylene for 1 h each using an automatic benchtop tissue processor.

*Note: The volume of xylene used is as stated above.*

**Caution:** Xylene is flammable and presents a severe fire hazard and is also toxic to the human body. Work only in designated areas and a fume hood. Avoid direct contact with reagents. Always wear gloves and other appropriate PPE.

9. Paraffin infiltration: Infiltrate the bones by three sequential incubations in molten paraffin (60 °C) baths for 1 h each using an automatic benchtop tissue processor.

*Note: The volume of paraffin used is as stated in step A7.*

**Caution:** Paraffin is flammable and poses a severe fire hazard. Work only in designated areas and a fume hood. Avoid direct contact with reagents. Always wear gloves and other appropriate PPE.

10. Paraffin embedding: Transfer the bones into embedding molds filled with molten paraffin using a tissue embedding station and allow them to set at room temperature for 1 h to ensure complete solidification.

11. Block removal and storage: After complete solidification, place the molds at -20 °C for 5–10 min to facilitate removal of the paraffin blocks containing the specimen.

**Pause point:** Paraffin blocks can be stored at room temperature or 4 °C for long-term storage.

12. Sectioning and slide labeling: Cut 4-μm sections using a microtome. Float the sections on a 42 °C water bath, then transfer them onto glass slides. Remove excess water by gently touching the section edges with absorbent paper and label the slides with a pencil.

**Caution:** Operating the microtome can present serious safety hazards, including risks from the sharp blade and crush/pinch injuries from mechanical components. Keep hands away from the blade path, use appropriate holders and tools for tissue section handling, and follow the instrument instructions strictly during operation. For first-time users, operate the microtome under the guidance of an experienced engineer or senior researcher.

13. Drying: Arrange the sections vertically on a slide rack and dry at room temperature for 1 h or in a 37 °C drying oven for 30 min, to ensure complete evaporation of residual moisture.

14. Dewaxing to storage: Dewax in a 60 °C oven for 2 h to remove paraffin from the bone sections and slides.

*Note: The purpose of this dewaxing step is to improve paraffin adhesion between the section and the slide, to remove as much surrounding paraffin as possible, and to thoroughly evaporate residual moisture to allow long-term storage of the same batch of sections at room temperature or 4 °C without mold.*

**Pause point:** Dewaxed sections can be stored at room temperature or 4 °C for long-term storage.

**Caution:** Paraffin is flammable and poses a severe fire hazard. Work only in designated areas and a fume hood. Avoid direct contact with reagents. Always wear gloves and other appropriate PPE.

**B. Silver nitrate staining of the osteocyte LCS**

1. Deparaffinization: Deparaffinize 4-μm paraffin sections by incubating in 100% xylene at room temperature for 10 min, followed by two sequential changes of 100% xylene for 10 min each, to ensure complete paraffin removal.

*Note: Section slides should be carefully selected under a microscope to ensure they contain complete bone tissue. For example, when using tibia or femur samples, the sections should ideally include the full proximal epiphysis, diaphysis, and distal epiphysis regions. In addition, please note that dewaxing in a 60 °C oven cannot completely remove paraffin from the slides or from the tissue itself. Therefore, prior to histological staining, the sections must be fully deparaffinized again in xylene and then rehydrated to ensure proper binding of staining reagents/antibodies and optimal staining results.*

**Caution:** Xylene is flammable and presents a severe fire hazard and is also toxic to the human body. Work only in designated areas and a fume hood. Avoid direct contact with reagents. Always wear gloves and other appropriate PPE.

2. Rehydration: Rehydrate the sections through a graded ethanol series (100%, 90%, 80%, and 70% ethanol) for 5 min each.

**Caution:** Ethanol is flammable and poses a severe fire hazard. Work only in designated areas and a fume hood. Avoid direct contact with reagents. Always wear gloves and other appropriate PPE.

3. Washing: Wash the sections twice with Milli-Q water, 5 min each.

*Note: Do not use tap water, PBS, TBS, or any salt-containing solutions, as they can interfere with subsequent silver nitrate staining. We place slides into different staining racks/jars depending on the number of slides used (e.g., a plastic jar that holds 5 slides or one that holds 24 slides). For thorough rinsing, use sufficient Milli-Q water to completely cover the tissue on the slides. For example, the 5-slide jar requires approximately 40 mL of Milli-Q water, whereas the 24-slide jar requires approximately 200 mL to ensure a thorough rinse.*

4. Preparation of silver nitrate staining solution: Prepare the silver nitrate staining solution rapidly and immediately before use as indicated in Recipe 3. Mix thoroughly. See General note 2.

5. Silver nitrate staining: After washing, quickly remove excess water around the tissue using absorbent paper. Place the slide horizontally on the bench and immediately apply the freshly prepared silver nitrate staining solution to the tissue section. Incubate by placing the slide horizontally on the lid of a water bath set to 50 °C (optional range: 50–70 °C) for 10 min. See General note 4.

6. Staining termination: After staining, rinse the slides twice with Milli-Q water for 5 min each to stop the staining reaction and remove excess staining solution.

*Note: Do not use tap water, PBS, TBS, or any salt-containing solutions, as they can react with silver nitrate and form flocculent precipitates, which will affect staining quality. We place slides into different staining racks/jars depending on the number of slides used (e.g., a plastic jar that holds 5 slides or one that holds 24 slides). For thorough rinsing, use sufficient Milli-Q water to completely cover the tissue on the slides. For example, the 5-slide jar requires approximately 40 mL of Milli-Q water, whereas the 24-slide jar requires approximately 200 mL to ensure a thorough rinse.*

7. Dehydration: Dehydrate the sections in 100% ethanol for 5 min.

*Note: In our dehydration procedure after staining, we directly immerse the stained sections in 100% ethanol for 5 min, without performing sequential (graded) dehydration. Based on our experience, this approach does not affect subsequent tissue morphology.*

**Caution:** Ethanol is flammable and poses a severe fire hazard. Work only in designated areas and a fume hood. Avoid direct contact with reagents. Always wear gloves and other appropriate PPE.

8. Clearing: Clear in 100% xylene for 10 min, and repeat three times.

**Caution:** Xylene is flammable and presents a severe fire hazard, and is also toxic to the human body. Work only in designated areas and a fume hood. Avoid direct contact with reagents. Always wear gloves and other appropriate PPE.

9. Removal of residual xylene: After clearing, place the slides in a fume hood for 5–10 min to allow residual xylene to evaporate.

**Caution:** Xylene is flammable and presents a severe fire hazard and is also toxic to the human body. Work only in designated areas and a fume hood. Avoid direct contact with reagents. Always wear gloves and other appropriate PPE.

10. Mounting: Apply approximately 20 μL of neutral balsam mounting medium to the bone section, then carefully place the coverslip, ensuring there are no air bubbles between the slide and the coverslip.

11. Drying prior to storage or LCS data acquisition: Dry the mounted slides for 12–18 h in a 37–50 °C oven to ensure complete solidification of the mounting medium. Then, use the slides immediately for LCS data acquisition under a microscope.

**Pause point:** After data acquisition, stained slides can be stored in slide boxes at room temperature for short-term storage.

## Validation of protocol

This novel and rapid protocol has been developed and validated in the following research article:

- Wu et al. [20]. A Rapid 10-Minute Silver Nitrate Staining Method for Visualizing the Osteocyte Lacuno-Canalicular System. *bioRxiv.*
  This protocol was validated as an efficient and effective method for staining the osteocyte LCS in mouse tibiae, producing clear LCS staining in cortical bone, the primary (1st) ossification center, and the secondary (2nd) ossification center (Figure 1).
  **Figure 1.**
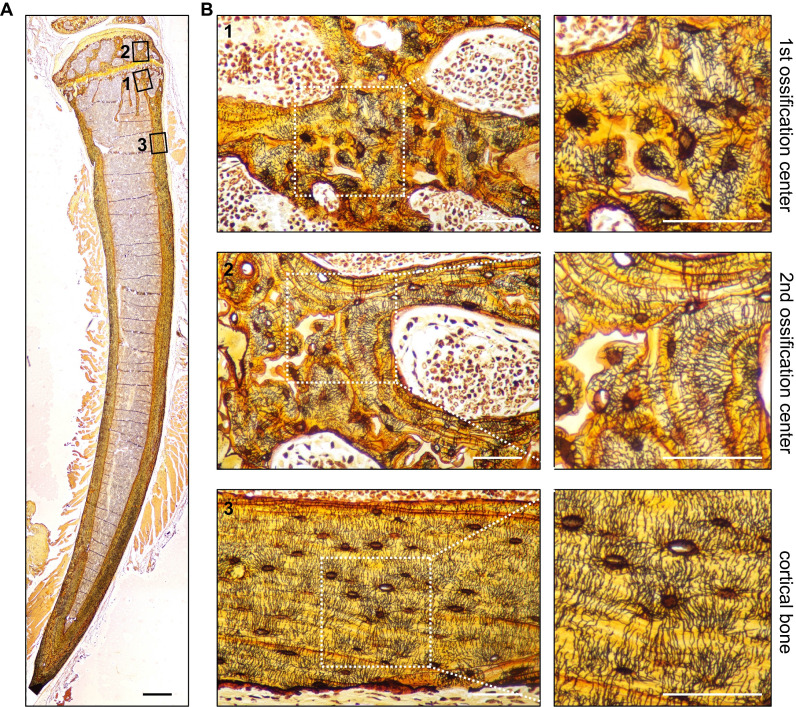
The Wu–Wang silver nitrate staining method enables rapid and efficient lacuno-canalicular system (LCS) staining in the mouse tibia. (A) Representative overview of silver staining of the osteocyte LCS in the tibia. Scale bar, 500 μm. (B) Higher-magnification images showing efficient staining of the osteocyte LCS in three different regions of the tibia. Scale bars, 50 μm. The numbers 1–3 indicate representative LCS-stained images from the first ossification center, the second ossification center, and the cortical bone region, respectively.
  This novel protocol enables rapid and effective LCS staining across multiple species, consistently yielding clear and reliable results (Figure 2), including in mammals (e.g., pig), amphibians (e.g., black-spotted frog), reptiles (e.g., Chinese three-keeled pond turtle), birds (e.g., domestic pigeon), and fish (e.g., zebrafish, rare gudgeon, grass carp, and Asian swamp eel).
  **Figure 2.**
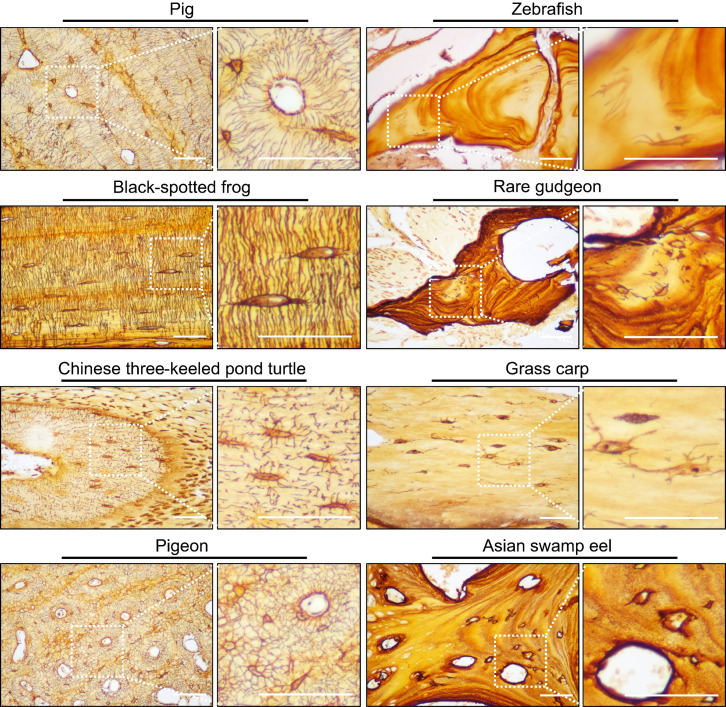
The Wu–Wang silver nitrate staining method enables rapid and efficient lacuno-canalicular system (LCS) staining in bones across multiple species. Scale bars, 50 μm.

## General notes and troubleshooting

**General notes**

1. Based on our observations, freshly prepared 2% (w/v) gelatin in 1% FA is clear and colorless. With increasing storage time—especially after 3 months—the solution gradually turns pale yellow. When a yellow gelatin solution is used to prepare the staining mixture together with 1 mol/L silver nitrate, staining efficiency decreases, and a longer incubation time may be required to obtain good LCS staining results (e.g., 15–20 min). Therefore, we recommend using this solution within 3 months. If the use of an over-stored solution is unavoidable, the staining time should be extended appropriately to achieve optimal staining.

2. The silver nitrate staining solution must be prepared in the dark. It should not be prepared in advance or stored. In our experience, a freshly prepared staining solution is clear and colorless, but it turns yellow to brown within 5–10 min and becomes dark brown after 1 h (Figure 3A). Darkened solutions do not produce effective LCS staining. Therefore, prepare the staining solution freshly and apply it immediately after mixing. For best results, apply the solution to bone sections on slides as quickly as possible, preferably within 5 min. For a paraffin section of a mouse knee joint containing both the femur and tibia, we typically use 300–400 μL of staining solution; adjust the volume proportionally for smaller or larger specimens.

3. As with many early-career researchers, our routine experimental practices were largely informed by published studies in bone–joint research laboratories and by the instructions provided by reagent manufacturers. However, even so, many papers and books still include vague statements, such as “10×, 15×, or 20× the volume of the tissue,” for fixation and decalcification [21–23]. Yet, in practice, it is rarely clarified what the actual tissue volumes are for different species and different bones (e.g., femur vs. tibia). Therefore, the meaning of numbers such as “10×” or “20×” can appear questionable, which may be why such descriptions confuse beginners in biological research. After an initial literature search, we were also surprised to find that definitive quantitative information was not readily available. To address this concern, we performed basic measurements on intact femurs and tibias from adult 2-month-old male C57BL/6J mice preserved in 75% ethanol. Specifically, we measured both mass and volume. For mass measurement, we weighed three femurs (n = 3) or three tibias (n = 3) and calculated the mean. For volume measurement, we used the following approach: first, 1 mL of Milli-Q water was added to an initial 1.5 mL Eppendorf (EP) tube. Then, three femurs or three tibias were added and fully immersed so that the water level increased. The displaced water was transferred by pipette to a second 1.5 mL EP tube to restore the water level back to the 1 mL mark of the first EP tube. Because the volume of 1 g of Milli-Q water is 1 mL, the mass of the transferred water was used to estimate the volume occupied by the bones. The measurements showed that, for an average femur from a 2-month-old mouse, the mean mass was ~0.0499 g, and the mean volume was ~0.0452 mL; for an average tibia, the mean mass was ~0.0397 g, and the mean volume was ~0.0340 mL. Therefore, we approximate that the volume of one complete femur is ~50 μL and that of one complete tibia is ~40 μL for a 2-month-old mouse. We can further estimate that the overall volume of one dissected femur and one dissected tibia from an adult mouse (after removing excess muscle and fat) is approximately 90–100 μL. Thus, when using the phrase “10-fold greater than the tissue volume,” we can roughly estimate that at least ~1 mL of fixative and decalcifying solution is required for one knee joint, i.e., including both a femur and a tibia. Therefore, during tissue fixation and decalcification, this information can be used as a reference to estimate the required volumes of fixative and decalcifying solution.

4. Ensure that the water bath is completely filled and that the water level fully contacts the lid of the water bath. This helps maintain a stable and uniform temperature at the lid surface, consistent with the set water temperature. Set the water-bath temperature in advance and ensure it has reached 50 °C before starting the incubation (optional temperature range: 50–70 °C). During incubation at 50–70 °C, the staining solution on the slide typically changes from clear and colorless to brownish-black within 10 min (Figure 3B). Alternatively, use a temperature-controlled incubator or a temperature-controlled hotplate. In our experience, during the 10-min incubation, there is no obvious evaporation whether the slide is placed on the water-bath lid or incubated in a temperature-controlled incubator.

5. We found that the staining quality of osteocyte LCS declined progressively with increasing decalcification time. After 10 min of silver staining, EDTA-decalcified paraffin sections from mouse bones decalcified for 4 weeks produced poorer LCS staining than sections decalcified for only 1 week (at EDTA concentrations of 0.5 mol/L or 10%/14%). However, when the staining time was extended to 20 min, all EDTA-decalcified samples (decalcified for 1, 2, 3, or 4 weeks) produced clear and effective LCS staining. In contrast, rapid decalcification solutions such as 5% FA can cause LCS structural disruption, making them poorly suited for LCS silver staining. Therefore, for LCS staining and analysis, we recommend using an EDTA-based decalcification solution. Decalcification for 7 days was sufficient to obtain well-prepared sections that could be stained efficiently and rapidly within 10 min using this method. When decalcification exceeds 7 days, we recommend increasing the silver staining time to 15–20 min, or alternatively increasing the incubation temperature from 50 to 60 °C to achieve efficient LCS visualization.

6. When staining LCS across different mouse ages under the same decalcification conditions (e.g., 0.5 mol/L EDTA for 7 days), the staining time required for optimal LCS visualization may vary slightly. For example, for 8-month-old mice, we found that incubation at 50 °C for 10 min was sufficient to obtain rapid, clearly stained sections. In contrast, 6-week- and 3-month-old mice required 20 min to achieve comparable staining clarity. This modest discrepancy likely reflects age-associated differences in bone mineralization. Specifically, after decalcification under identical conditions, younger bones may retain fewer residual mineral salts than more mature bones, which could slightly affect the efficiency of the rapid LCS silver staining method. Therefore, when analyzing and comparing LCS densities in paraffin sections from mice of different ages, we recommend extending the silver staining time to 15–20 min or, alternatively, increasing the incubation temperature from 50 to 60 °C, to achieve clear and reliable visualization.

7. The properties and staining performance of type-A and type-B gelatin vary substantially across different commercial brands and manufacturers. In our previous studies [15–16], we found that type-A gelatin is generally unsuitable for silver nitrate staining of LCS because it produces abundant black, granular precipitates on bone sections, rendering LCS data unusable. In our recent work [20], we further observed marked differences among type-A gelatins from different brands. For example, Sangon^TM^ type-A gelatin is not suitable for silver nitrate staining because it generates a large amount of black granular material on bone tissue after staining. In contrast, certain type-A gelatins from Yuanye^TM^ and Sigma^TM^ (specific product lots/catalog numbers) can be used for silver nitrate staining without producing such black granules. For type-B gelatin, we tested four brands and found that all supported silver nitrate staining for LCS and did not produce black granular precipitates. However, the intrinsic physicochemical properties of gelatin—such as its isoelectric point, pH, gel strength, and other factors—may directly influence silver staining performance. Importantly, we also found that staining efficiency differed among type-B gelatins: Sangon^TM^ type-B gelatin produced clear LCS staining only within 5 min, whereas the other three (Solarbio^TM^, Yuanye^TM^, and Sigma^TM^) required 10 min to achieve comparable clarity. Therefore, when an appropriate type-B gelatin brand is used, the LCS staining time can be further reduced to within 5 min. Additionally, fish-derived gelatin did not produce effective LCS staining in our tests. Overall, we recommend prioritizing type-B gelatin for preparing the LCS silver staining solution.

**Figure 3.**
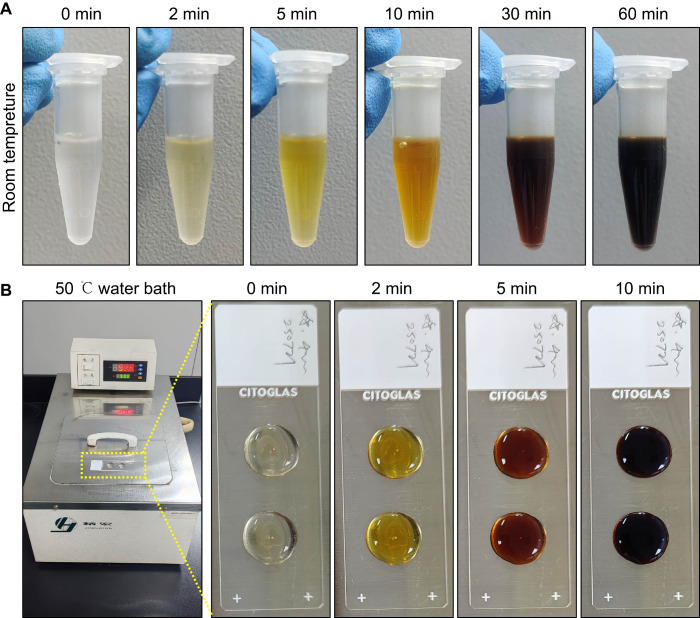
Time-dependent color changes of the silver nitrate staining solution during the 10-min staining procedure at (A) room temperature and (B) at 50 °C
